# Identification of the mRNA Expression Status of the Dopamine D2 Receptor and Dopamine Transporter in Peripheral Blood Lymphocytes of Schizophrenia Patients

**DOI:** 10.1371/journal.pone.0075259

**Published:** 2013-09-25

**Authors:** Liang Liu, Guozhen Yuan, Zaohuo Cheng, Guofu Zhang, Xiaowei Liu, Huifang Zhang

**Affiliations:** Clinical Psychiatry Department, Wuxi Mental Health Center of Nanjing Medical University, Wuxi, China; School of Medicine and Health Sciences, University of North Dakota, United States of America

## Abstract

The aim of this study was to detect the mRNA expression levels of the dopamine D2 receptor (DRD2) and dopamine transporter (DAT) in peripheral blood leukocytes (PBLs) of schizophrenia patients and to explore the relationship between the mRNA expression levels and the clinical symptoms of schizophrenia. The research included 25 cases of acute schizophrenia patients, 27 cases of chronic schizophrenia patients, and 30 healthy controls. In every case, we measured the mRNA levels of DRD2 and DAT in PBLs by real-time quantitative reverse transcription-polymerase chain reaction (real-time RT-PCR), and we evaluated the patients’ clinical symptoms using the Positive and Negative Syndrome Scale (PANSS). DRD2 mRNA levels in PBLs of acute schizophrenia patients, chronic schizophrenia patients, and healthy controls were 0.32±0.13, 0.37±0.19, and 0.34±0.09, respectively, and the difference was not significant. DAT mRNA levels in PBLs of the abovementioned groups were 0.48±0.24, 0.58±0.21 and 0.39±0.24, respectively (F = 4.330, P = 0.017), and comparisons between every group showed that DAT mRNA levels in PBLs of chronic schizophrenia patients were significantly higher than those in healthy controls (MS interclass = 0.198, p = 0.005). The correlation between DRD2 mRNA levels in PBLs and the positive symptom points of PANSS in acute schizophrenia patients was significant (r = 0.443, p = 0.044). In conclusion, DRD2 mRNA levels in PBLs are correlated with positive symptoms in acute schizophrenia patients, and DAT mRNA levels in PBLs of chronic schizophrenia patients are over-expressed.

## Introduction

Schizophrenia is a severe, chronic mental disorder, and it affects approximately 1% of the world’s population [1]. For individuals who have a schizophrenic relative in her/his family, the chances of the individual having schizophrenia raises to 65–85% [2]. The etiology and pathophysiology of schizophrenia remain obscure, and its current diagnosis is based on complex clinical symptoms. The application of easily detectable peripheral molecular markers could substantially help the diagnosis of psychiatric disorders [3].

The dominant “dopamine hyperfunction hypothesis” was supported by molecular, pharmacological and clinical evidence for over 40 years [4]–[6]. Many of the signs and symptoms of schizophrenia can be reproduced in humans or animal models with dopaminergic drugs [7], [8]. In addition, a recent meta-analysis was performed that was based on over 1000 association studies on schizophrenia, and this study highlighted 16 genes, which were mostly dopamine-related, including catechol-O-methyltransferase (COMT) and dopamine receptors D1, D2, and D4 (DRD1, DRD2 and DRD4) [9].

Dopamine (DA) is a monoamine catecholamine neurotransmitter that acts through its D1 and D2 classes of receptors present in the target cells [10]. The D1 class of receptors includes the D1 and D5 subtypes, which increase intracellular cAMP on activation [10]. In contrast, the D2 class of receptors, which includes D2, D3 and D4 subtypes, inhibit intracellular cAMP on stimulation [10]. DA reuptake mostly depends on the presence and activity of the DA transporter (DAT), a 80 kD glycoprotein belonging to the large Na+/Cl− dependent transporter family, which includes norepinephrine, serotonin, GABA and glycine transporters [11].

The presence of all five dopamine receptor genes (DRD1, DRD2, DRD3, DRD4, DRD5) has been reported in normal human leukocytes by several studies using receptor binding assay or reverse transcription-polymerase chain reaction (RT-PCR) methods [12]–[16]. DAT expression was also detected in lymphocytes and shows similar patterns to that seen in the brain [17], [18]. In previous studies, we reported that tyrosine hydroxylase (TH), the dopamine synthesis rate-limiting enzyme, is over-expressed in peripheral blood leukocytes (PBLs) of drug-free schizophrenia patients [19].

Dopamine receptors are the key elements of the dopaminergic system. The dopamine receptors in PBLs may reflect the status of homologous brain receptors [11], [20], [21]. Analysis of dopamine receptors in PBLs is a useful tool for evaluating the functional properties of dopaminergic function that underlie the variation in complex psychological and psychopathological traits [6], [13], [22].

The changes of the DA system in PBLs of schizophrenia have been mentioned, and a review showed that most research has focused on DRD3 [11]. However, the gene expression levels of DRD2 and DAT in PBLs of schizophrenia patients and their relationship with clinical symptoms have not been reported in many studies. DRD2 is the main target of antipsychotic drugs action, for both the classical antipsychotic drugs and the second generation antipsychotic drugs. DAT is significantly relevant to the adverse reaction of antipsychotic drugs. A recent study demonstrated the reduction of DAT mRNA levels in PBLs of psychotic patients with respect to healthy subjects [23]. DRD2 mRNA levels in PBLs of drug-naive schizophrenia patients were found to be over-expressed in a microarray analysis [3].

Based on the above studies, our present study aimed to identify a new profile of peripheral biomarkers of schizophrenia by characterizing the expression patterns of DRD2 and DAT in PBLs of acute schizophrenia patients and chronic schizophrenia patients and to explore the relationship between their mRNA levels and the psychopathological status of schizophrenia as evaluated by the Positive and Negative Symptom Scale (PANSS).

## Materials and Methods

### Participants

Patients were recruited from the inpatient unit of the Department of Psychiatry, Wuxi Mental Health Center of Nanjing Medical University from April-October 2010. Each patient went through profound medical examination by a senior psychiatric attending physician, including physical and neurological examination, as well as routine laboratory tests, and fulfilled the criteria for schizophrenia according to the Diagnostic and Statistical Manual-IV (DSM-IV) [24]. The group of acute schizophrenia patients included 25 individuals with an illness duration of less than 1.5 years [24] who were first-episode, drug-naive schizophrenia patients or recurrent, drug-free schizophrenia patients with self-withdrawn antipsychotic drugs for more than 1 month. The group of chronic schizophrenia patients included a total of 27 cases with an illness duration greater than 2 years [25] and were residual schizophrenia patients [24], [25] or deteriorated schizophrenia patients [25], according to the criteria of chronic schizophrenia, residual schizophrenia, and deteriorated schizophrenia in the Chinese Classification and Diagnostic Criteria of Mental Disorders Version 3 (CCMD-3) [25] and the criteria of the residual type of schizophrenia in the DSM-IV [24]. The chronic schizophrenia patients had been treated by more than two antipsychotic drugs with long-term treatment of single clozapine for more than 6 months (drug dose conforms to the therapeutic dose). Thirty staff members from the Wuxi mental health center who volunteered to participate comprised the healthy control group.

All of the subjects were Han people, without any previous history of substance abuse, neurological disease, immunologic disease, or major surgeries. The control group members had no history of neuropsychological disease. The female subjects were not pregnant, lactating or menstruating at the time of the study. The individuals in all three groups had no significant difference in the subsets, divided by gender and age. The demographic and clinical characteristics of the subjects are shown in Table 1.

**Table 1:** Demographic and clinical characteristics of subjects.

	Acute patients	Chronic patients	Controls
	(N = 25)	(N = 27)	(N = 30)
Mean age (S.D.)	32.63 (10.21)	31.33 (9.85)	30.81 (10.36)
Sex (Male) (%)	14 (56.00)	15 (55.56)	15 (50.00)
Duration of illness (months) (S.D.)	8.93 (3.44)	>24	NA
PANSS Total scores mean (S.D.)	95.82 (10.74)	70.32 (9.57)	NA

The individuals in all three groups had no significant difference in gender (X2 = 0.256, P = 0.880) or age (F = 0.228, P = 0.797).

Evaluation of the PANSS was performed through an oral case history interview and psychiatric interview on the day that blood was drawn.

#### Ethics statement

This study was conducted according to the principles expressed in the Declaration of Helsinki. Approval was given by the ethics committee of the Wuxi mental health center. Prior to the study, procedures were fully explained, and written informed consent was obtained from each subject or the fist legal guardian of patients with limited/no disposing capacity.

### Methods

#### Sample collection, cell separation and RNA isolation

2 ml of fasting ulnar vein blood were drawn from each subject and collected in vacutainer tubes containing preservative-free heparin. PBLs were isolated by standard density centrifugation using 10 ml of lymphocyte isolation solution (d = 1.083, Shanghai Biochemical Reagents, Inc.). Total ribonucleic acid (RNA) was extracted with the SV Total RNA Isolation System (Promega Co. USA), which incorporated a DNase treatment step. The purity and content were determined with an optical density ratio of 260 nm to 280 nm (1.8∼2.1) by ultraviolet spectrophotometry. The integrity was assessed by denaturing agarose gel electrophoresis, showing an approximate ratio of 2∶1 of 28 s to 18 s bands by ethidium bromide staining.

RNA extraction and reverse transcription were performed 3 hours after the blood sample was drawn to minimize RNA degradation. To minimize the following batch effects, time, procedure, and lab technician performing the RNA extraction and reverse transcription were unified. The fasting blood was drawn at 6∶00 am, and the RNA was extracted and reverse-transcripted by the same lab technician with the same experimental procedure.

#### Reverse Transcription-Polymerase Chain Reaction (RT-PCR) and relative quantitative analysis

The first cDNA was synthesized from total RNA by the AMV reverse transcriptase kit (Promega Co. USA) using Oligo d(T)15 as primer. The reaction system contains 400 Ng total RNA template, 5 µl 5×reaction buffer, 1.25 µl dNTP (10 mM), 1 µl Oligo d(T)15 (0.5 µg/µl), 1 µl AMV enzyme (200 U/µl) and nuclease-free water up to 25 µl. The reaction mixture was placed in a 42°C water-bath for 60 min and a 90°C water-bath for 5 min to end the reaction, and it was stored at −70°C.

The primer sequences of glyceraldehyde-3-phosphate dehydrogenase (GAPDH), DRD2 and DAT were designed with Primer-Blast software on the United States National Center for Biotechnology Information (NCBI) website. The locations of all primer sequences were selected to avoid the effect of alternative splicing and are shown in Table 2. All primers were synthesized by Songon Co. (Shanghai, China).

**Table 2:** The primer sequences of GAPDH, DRD2 and DAT.

Gene	Primer sequences	Location	Size
GAPDH	For: 5′-ACGGATTTGGTCGTATTGGG-3′	Exon 4/5	214 bp
	Rev: 5′-CGCTCCTGGAAGATGGTGAT-3′	Exon 5/6	
DRD2	For: 5′-AGACCATGAGCCGTAGGAAG-3′	Exon 7	96 bp
	Rev.: 5′-GCAGCCAGCAGATGATGA-3′	Exon 8	
DAT	For: 5′-CGGCCAGACCAAGAGGGAAGAAGCA-3′	Exon 1/2	53 bp
	Rev: 5′-TGGGCACACTGGGAGTTGAGGAA-3′	Exon 2	

A real-time polymerase chain reaction (real-time PCR) was carried out on the ABI Prism 7500 PCR instrument (ABI Co., USA) using Qiagen QuantiTect SYBR Green PCR Kit (Qiagen Co., USA), and every sample was carried out using parallel detection 3 times to obtain the average Ct value. The Reaction system was as follows: cDNA template 4 µl, 12.5 µl of 2×QuantiTect SYBR Green PCR Master Mix, upstream and downstream primers of GAPDH, DRD2 or DAT (10 µM) 1 µl, and adding water to reach 25 µl. The reaction conditions were as follows: 95°C pre-denaturing for 15 min, 94°C denaturation for 15 s, 57°C annealing for 30 s and extension at 72°C for 30 s, cycling 40 laps.

The DAT and DRD2 humanized full length transcription cDNA clones, purchased from Fulengen Co.(Guangzhou, China), were diluted in gradient 10-fold as templates. According to the above reaction system and reaction conditions for amplification, we drew the standard curve with the slope and intercept calculated by the corresponding cycle threshold value (Ct value), and we analyzed the relative quantitation of DRD2 and DAT mRNA expression levels using a standard curve method with GAPDH as the internal control. To minimize the batch effects of gene expression analysis, we standardized the experiment process, such as time, procedure, and lab technician. We performed repeatedly the standard curve of target genes in every reaction plate, and set the same threshold value of every target gene.

#### Statistical analysis

Statistical determinations were performed using statistical software (SPSS version 17). The homogeneity test of variance was firstly taken among the mRNA levels of two target genes in three groups. If the variance was equal, the One-way ANOVA and least significant difference (LSD) methods were chosen. If the variance was not equal, the Welch approximate analysis of variance and Tamhane methods were chosen. The PANSS score was not a continuous variable, so the Spearman’s correlation analysis was utilized to analyze the relationship between the mRNA levels of two target genes and the PANSS scale score. The results are expressed as the mean±standard deviation (SD).

## Results

### The Standard Curves of GAPDH, DRD2 and DAT

**Figure 1:**
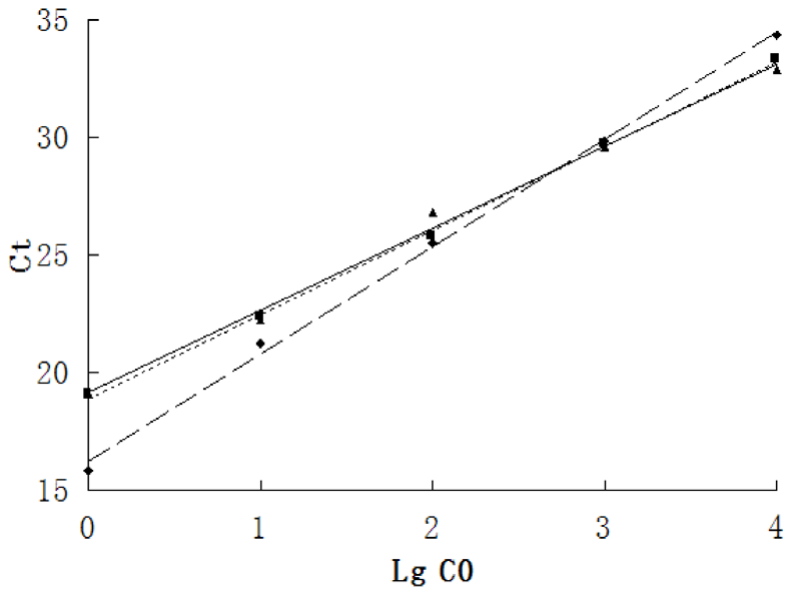
Standard curves of GAPDH, DAT1 and DRD2 mRNA. The standard curves of real-time PCR of GAPDH, DAT1 and DRD2 cDNA diluted in gradient 10-fold showed a good linear relationship. ▴:DAT, R^2^ = 0.994, y = 3.4844x+19.165; ♦:DRD2, R^2^ = 0.998, y = 4.5613x+16.226; ▪:GAPDH, R^2^ = 0.998, y = 3.5773x+18.880; Ct value: Cycle threshold value; LgC0: Logarithm of the initial concentration.

The DAT and DRD2 humanized full length transcription cDNA clones were diluted in gradient 10-fold as templates, and the standard curves were drawn with the slope and intercept calculated by the corresponding Ct value in the amplification. The standard curves of GAPDH, DRD2 and DAT all showed a good linear relationship (Figure 1), and therefore, we could analyze the relative quantitation of DRD2 and DAT mRNA expression levels using the standard curve method with GAPDH as the internal control.

### Comparisons of DAT and DRD2 mRNA Levels between the Three Groups

**Figure 2:**
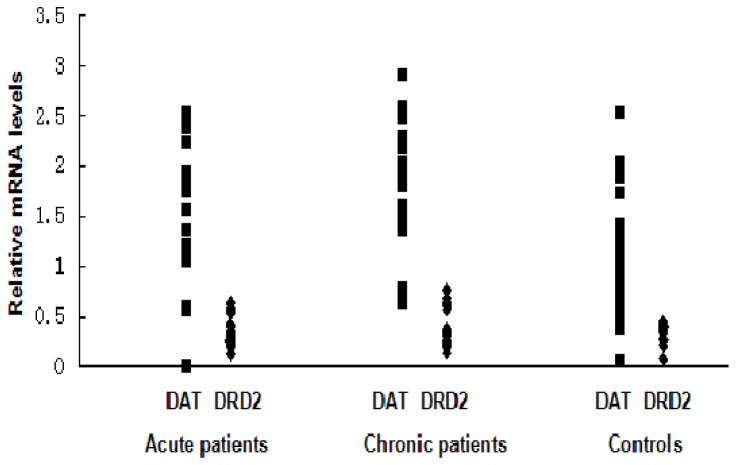
DAT and DRD2 mRNA levels between the three groups. DAT mRNA levels of the chronic schizophrenia patients were significantly higher than those in the control group (MS interclass = 0.198, p = 0.005), No significant difference was found in DRD2 mRNA levels between the three groups. ▪:DAT; ♦:DRD2.

DAT mRNA levels in the PBLs demonstrated significant differences (F = 4.330, p = 0.017) among the three groups. As the comparisons between every group showed, the DAT mRNA levels of the chronic schizophrenia patients were significantly higher than those in the control group (MS interclass = 0.198, p = 0.005). However, there was no significant difference in the DAT mRNA levels between the acute schizophrenia patients and the healthy controls, or between the acute schizophrenia patients and the chronic schizophrenia patients. No significant differences were found in the DRD2 mRNA levels among the three groups (F = 0.510, P = 0.605) (Table 3, Figure 2).

**Table 3:** Comparisons of DAT and DRD2 mRNA levels between the three groups (Mean±SD).

	Acute patients	Chronic patients	Controls	F	P-value
	(N = 25)	(N = 27)	(N = 30)		
DAT	0.48±0.24	0.58±0.21▴	0.39±0.24	4.330	0.017*
DRD2	0.32±0.13	0.37±0.19	0.34±0.09	0.510	0.605

In the homogeneity test of variance, the Levene value of DAT mRNA levels in the three groups was 0.37 (p = 0.692), and the One-way ANOVA and LSD methods were chosen; the Levene value of DRD2 mRNA levels in the three groups was 4.89 (p = 0.011), and the Welch approximate analysis of variance and Tamhane methods were chosen.

* Comparisons between the three groups, p = 0.017, <0.05;

▴ Comparison between the chronic schizophrenia patients and healthy controls, MS interclass = 0.198, p = 0.005, <0.05.

### Correlation Analysis between DAT and DRD2 mRNA Levels and PANSS Scores in Schizophrenia Patients

**Figure 3:**
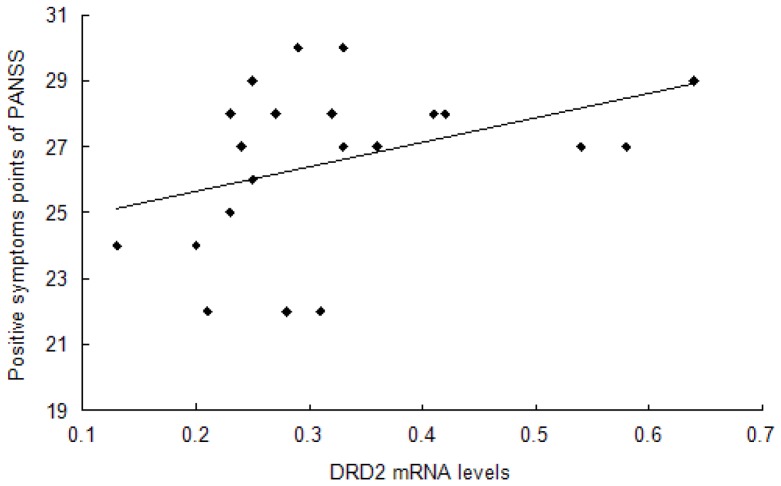
Relationship between DRD2 mRNA levels and positive symptom points of PANSS in acute patients. Spearman’s correlation analysis was chosen to find the significant relationship between the mRNA levels of DRD2 and positive symptom points of the PANSS score (r = 0.443, p = 0.044).

In acute patients, a significant relationship was found between the DRD2 mRNA level and positive symptom points of the PANSS (r = 0.443, p = 0.044) (Table 4, Figure 3). However, the relationship between the DAT mRNA level and PANSS total scores (r = −0.418, p = 0.075), or the general pathological symptom points of PANSS (r = −0.434, p = 0.063), were not significant (Table 4). In chronic patients, no significant relationship was found between the DRD2 and DAT mRNA levels and PANSS scores (Table 4).

**Table 4:** Correlation analysis between DAT and DRD2 mRNA levels and PANSS scores in schizophrenia patients (r value).

	Acute patients		Chronic patients	
	DAT	DRD2	DAT	DRD2
PANSS total scores	−0.418	−0.116	−0.387	−0.156
Positive symptoms points	−0.245	0.443*	−0.255	0.412
Negative symptoms points	0.018	−0.114	0.021	−0.205
General pathologicalsymptoms points	−0.434	−0.152	−0.399	−0.117

* p = 0.044, <0.05.

### Analysis of Clinical Factors for DAT and DRD2 mRNA Levels

A correlation analysis was performed to analyze the relationship between DAT and DRD2 mRNA levels and gender and age in all subjects, or the duration of illness in schizophrenia patients, but no statistically significant difference was found (Table 5).

**Table 5:** Influence of clinical factors on DAT and DRD2 mRNA levels.

	Gender	Age	Duration
DRD2	NS	NS*	NS*
DAT	NS*	NS*	NS*

NS*: Not statistically significant.

## Discussion

As all known, the pathogenesis research of schizophrenia has consistently focused on the “dopamine hyperfunction hypothesis”. Meanwhile, a lot of research also indicates that DA is an important regulator of normal immunity [26], and changes in the concentrations and/or receptors of DA are responsible for abnormal immune functions seen in schizophrenia patients and those with Parkinson’s disease [27]–[31]. The dopamine receptors in PBLs may reflect the status of homologous brain receptors [20], [21], and five types of DR, D1 through D5, are co-expressed at different levels and in various combinations in the CNS and peripheral tissues [13]. A review has also supported the feasibility of PBLs as a cellular tool with which to investigate DA derangement in neuropsychiatric disorders [11], particularly because of the economic and technical difficulties of investigating such changes directly in the CNS in-vivo. Based on the above research foundation, this study aimed to detect mRNA expression levels of DRD2 and DAT in PBLs of schizophrenia patients by RT-PCR methods and analyzed the relationship between the schizophrenia patients’ mRNA levels and PANSS scores.

Our research found no significant differences in the DRD2 mRNA levels between the three groups. Although Zvara’s research reported the over-expression of DRD2 mRNA in PBLs of 13 drug-naive/drug-free schizophrenia patients [3], Yao’s research also found that the difference of DRD2 mRNA levels in PBLs was not statistically significant between 30 first-time hospitalized schizophrenia patients and 26 healthy controls [32], which is similar to our finding. Therefore, DRD2 mRNA levels in PBLs might be a state index related to psychiatric symptoms, but not a quality index related to schizophrenia. Schizophrenia is a group of syndromes with great heterogeneity, and the symptoms of schizophrenia patients with the same diagnosis can have vast differences (such as positive symptoms and negative symptoms) [33]. It may be appropriate that schizophrenia patients are distinguished according to their characteristics of psychiatric symptoms to avoid the confusion of such problems when research is conducted. In addition, the acute schizophrenia group had some recurrent schizophrenia patients with antipsychotic drugs self-withdrawn for more than one month, and the drug’s long-term legacy effect could not be excluded.

We found that the DAT mRNA levels in PBLs have significant differences between the three groups. As comparisons between every group showed, the DAT mRNA levels of the chronic schizophrenia patients were significantly higher than those of the control group. However, there was no significant difference found in the DAT mRNA levels between the acute schizophrenia patients and the healthy controls, or between the acute schizophrenia patients and the chronic schizophrenia patients. We only found the related brain imaging studies of DAT in schizophrenia patients, but no DAT mRNA expression studies were found in PBLs of schizophrenia patients. Our results of acute schizophrenia patients were consistent with brain imaging studies. There was no evidence indicating altered density of striatal DAT in schizophrenia in a meta-analysis that included thirteen single photon emission tomography (SPECT) or positron emission tomography (PET) studies in schizophrenia [34] and a 4-year follow-up study on SPECT of DAT in neuroleptic-naive first episode schizophrenia patients [35]. In terms of the medicated chronic schizophrenia patients, data were not consistent. SPECT research has found that chronic schizophrenia patients treated with classic antipsychotic drugs showed a 36–63% increase in DAT binding sites compared with healthy volunteers [36]. However, a PET study found that DAT densities in medicated chronic schizophrenia patients were significantly lower than those in controls, both in the caudate and the putamen (−9 to −16%) [37]. The group of chronic schizophrenia patients in our study consisted of residual schizophrenia patients or deteriorated schizophrenia patients with an illness duration of greater than 2 years [24], [25], and they had been treated by more than two antipsychotic drugs, as well as with a long-term treatment of single clozapine for greater than 6 months (drug dose conforms to the therapeutic dose). Thus, according to our results, we inferred that long-term antipsychotic treatment may have altered the mRNA levels of the presynaptic membrane DAT of chronic schizophrenia patients. This effect needs to be further confirmed by a rigorous control study.

As In terms of the relationship between mRNA levels of target genes in PBLs and the PANSS scores, we found a significant relationship between the DRD2 mRNA level and positive symptom points of PANSS in acute patients. This result can echo the “dopamine function hyperfunction hypothesis” of schizophrenia and the idea from psychopharmacology that mutual verification of antipsychotic drugs controls the positive symptoms of schizophrenia through their effects on the DRD2 receptor. There are few reports about the relevance between DRD2 mRNA levels in PBLs and the psychiatric symptoms of schizophrenia. We also found that the relationship between the DAT mRNA level and PANSS total scores (r = −0.418, p = 0.075), or the general pathological symptom points of PANSS (r = −0.434, p = 0.063), were not significant in acute patients. Increasing the sample size may make the results much clearer.

In chronic schizophrenia patients, the abovementioned type of relationship between DRD2 mRNA levels and positive symptom points of PANSS was not found. The chronic patients were residual schizophrenia patients or deteriorated schizophrenia patients with an illness duration of greater than 2 years [24], [25], and their PANSS total scores were only 70.32±9.57, which were significantly lower than the acute schizophrenia group, whose scores were 95.82±10.74. The relatively lower PANSS scores with smooth fluctuations in the chronic patients might affect this relationship. We also found no significant relationship between the DAT mRNA level and PANSS scores. The feature of PANSS scores in chronic patients mentioned above and the overexpression of DAT mRNA levels in chronic schizophrenia patients might explain this result.

Taken together, therefore, we found that the DRD2 mRNA levels in PBLs were correlated with positive symptoms in acute schizophrenia patients, and DAT mRNA levels in PBLs of medicated chronic schizophrenia patients were over-expressed. It is well-known that the analysis of dopamine receptors in PBLs is a useful tool for evaluating the functional properties of dopaminergic function that underlie the variation in complex psychological and psychopathological traits [6], [13], [22]. However, some limitations to the use of PBLs as possible biomarkers for studying the changes of the DA system in neuropsychiatric diseases should be acknowledged. When these cells are surrounded by different environments, they are subjected to different regulatory mechanisms [38]. To confirm these genes as potential biomarkers for a schizophrenia diagnosis, a high-throughput analysis of the interaction of already reported molecular markers and genes related to schizophrenia is needed.
